# Effectiveness of Abbott BinaxNOW Rapid Antigen Test for Detection of SARS-CoV-2 Infections in Outbreak among Horse Racetrack Workers, California, USA

**DOI:** 10.3201/eid2711.211449

**Published:** 2021-11

**Authors:** Krishna Surasi, Kristin J. Cummings, Carl Hanson, Mary Kate Morris, Maria Salas, David Seftel, Liza Ortiz, Ruwan Thilakaratne, Cameron Stainken, Debra A. Wadford

**Affiliations:** Centers for Disease Control and Prevention, Atlanta, Georgia, USA (K. Surasi);; California Department of Public Health, Richmond, California, USA (K. Surasi, K.J. Cummings, C. Hanson, M.K. Morris, M. Salas, R. Thilakaratne, C. Stainken, D.A. Wadford);; Golden Gate Fields, Berkeley, California, USA (D. Seftel);; City of Berkeley Public Health Officer Unit, Berkeley (L. Ortiz);; Kaiser Permanente San Francisco Internal Medicine Residency Program, San Francisco, California, USA (C. Stainken);; University of California San Francisco School of Medicine, San Francisco (C. Stainken).

**Keywords:** COVID-19, coronavirus disease, SARS-CoV-2, severe acute respiratory syndrome coronavirus 2, viruses, respiratory infections, zoonoses, SARS-CoV-2 RT-PCR testing, SARS-CoV-2 antigen testing, workplace, disease outbreaks

## Abstract

The Abbott BinaxNOW rapid antigen test is cheaper and faster than real-time reverse transcription PCR (rRT-PCR) for detecting severe acute respiratory syndrome coronavirus 2. We compared BinaxNOW with rRT-PCR in 769 paired specimens from 342 persons during a coronavirus disease outbreak among horse racetrack workers in California, USA. We found positive percent agreement was 43.3% (95% CI 34.6%–52.4%), negative percent agreement 100% (95% CI 99.4%–100%), positive predictive value 100% (95% CI 93.5%–100%), and negative predictive value 89.9% (95% CI 87.5%–92.0%). Among 127 rRT-PCR–positive specimens, the 55 with paired BinaxNOW-positive results had a lower mean cycle threshold than the 72 with paired BinaxNOW-negative results (17.8 vs. 28.5; p<0.001). Of 100 specimens with cycle threshold <30, a total of 51 resulted in positive virus isolation; 45 (88.2%) of those were BinaxNOW-positive. Our comparison supports immediate isolation for BinaxNOW-positive persons and confirmatory testing for negative persons.

Rapid antigen tests, such as Abbott BinaxNOW (https://www.abbott.com) test kits, offer a less expensive and faster alternative to nucleic acid amplification tests, such as real-time reverse transcription PCR (rRT-PCR), in the diagnosis of coronavirus disease (COVID-19) (*1*,*2*). Previous studies of BinaxNOW compared with rRT-PCR have demonstrated a high negative percent agreement (NPA) (99.4%–100%) but variable positive percent agreement (PPA) (52.5%–89.0%). Performance was better among symptomatic persons, specimens with cycle threshold (C_t_) <30 (suggestive of higher viral loads), and specimens with positive viral cultures (*3*–*8*). These reports have focused on community testing sites and outbreaks in healthcare facilities.

Throughout the pandemic, certain nonhealthcare occupational groups (e.g., meat and poultry processing workers) have experienced higher risk of contracting COVID-19; this higher risk is attributable to workplace hazards, such as lack of appropriate personal protective equipment, densely populated work areas, poorly ventilated workspaces, and prolonged close contact (*9*,*10*). These workplaces might benefit from effective rapid antigen tests that enable employers to quickly identify persons infected with severe acute respiratory syndrome coronavirus 2 (SARS-CoV-2) for isolation and to guide contact tracing, thereby reducing workplace transmission. Despite the need for research on this topic, information on the performance of BinaxNOW in the setting of nonhealthcare workplace outbreaks is lacking.

During October 20, 2020–January 15, 2021, a horse racetrack (the facility) in California, USA, experienced a COVID-19 outbreak among its 563 employees and independent contractor workers (hereafter collectively called facility staff). Nearly half (n = 278; 49.4%) of the staff lived onsite in facility-provided housing, and many performed essential duties (e.g., grooming, feeding) related to the basic care of the >1,100 horses stabled there.

The outbreak was discovered by the contact tracing efforts of the local health department (LHD), the City of Berkeley Public Health Officer Unit. In response, the LHD ordered that all nonessential work activities (e.g., horse racing) be stopped until mass testing of all staff demonstrated no further transmission. The initial round of rRT-PCR testing (round 0) occurred on November 14–15, 2020, and identified 169 SARS-CoV-2–positive staff who were subsequently isolated. At this time, all staff were assumed to have been exposed. Those living onsite were moved to hotel rooms to quarantine, and those living offsite quarantined in their homes. No staff were permitted to return to onsite residence until the outbreak had ended. However, some quarantined employees were permitted to return to work if they were needed to perform duties related to essential care of the horses. Additional rounds of testing were needed to monitor ongoing transmission and determine when the outbreak had ended. The LHD decided to use BinaxNOW as a supplement to rRT-PCR to more quickly identify SARS-CoV-2–positive employees for isolation. This use provided an opportunity to assess the effectiveness of the BinaxNOW rapid antigen test in detecting SARS-CoV-2 infection in a nonhealthcare workplace outbreak. The purpose of this analysis is to compare BinaxNOW with rRT-PCR in paired specimens from persons during a COVID-19 outbreak among horse racetrack workers. These findings could inform testing protocols used to contain future outbreaks of COVID-19 in nonhealthcare workplaces.

## Methods

The facility, in collaboration with the LHD and the California Department of Public Health (CDPH) laboratory, conducted 6 rounds of serial testing of its staff with paired BinaxNOW rapid antigen and rRT-PCR tests during November 25–December 22 (rounds 1–6). Testing frequency was determined by the LHD and changed as the outbreak progressed. Each round was intended to test all staff who had not yet tested positive by BinaxNOW or rRT-PCR to continue identifying potentially infectious persons. Staff who tested positive by either BinaxNOW or rRT-PCR were isolated and excluded from further testing.

All specimen collection and antigen testing occurred outdoors in the parking lot of the facility. On the day of testing, a facility administrative employee conducted registration and collected demographic data, including self-reported race and ethnicity. Symptom information was elicited by asking staff if they were experiencing any COVID symptoms, such as fever, headache, or loss of taste. Bilateral anterior nasal swab specimens were collected by either the racetrack physician or one of the racetrack veterinarians trained in collection procedures. A first swab specimen was used for onsite BinaxNOW testing; a second swab specimen was placed in viral transport medium and chilled on ice packs before transport to the CDPH laboratory for rRT-PCR testing 24–72 hours after collection. All specimens in viral transport medium were frozen at –70°C within 12 hours of delivery to the laboratory. BinaxNOW test results were interpreted immediately at the 15-minute read time by the racetrack physician in accordance with the test kit instructions, along with the updated scoring criteria described by Pilarowski et al. (*5*), which indicates that bands are scored as positive only if they extend across the full width of the strip, irrespective of the intensity of the band. Because BinaxNOW testing was not performed for round 0, those 169 rRT-PCR–positive specimens were not included in this analysis.

For rRT-PCR, we isolated and purified viral nucleic acid (NA) from the swab specimens by using the KingFisher Flex Purification System and the MagMAX Viral/Pathogen Nucleic Acid Isolation Kit (ThermoFisher Scientific, https://www.thermofisher.com). We performed rRT-PCR by using the ThermoFisher TaqPath COVID-19 Combo Kit, which targets 3 SARS-CoV-2 viral regions (nucleocapsid protein gene, spike protein gene, and open reading frame 1ab), and the Applied Biosystems 7500 Fast Dx Real-Time PCR Instrument (ThermoFisher Scientific), according to the manufacturer’s instructions. Real-time RT-PCR–positive specimens with C_t_ <30 were also cultured for SARS-CoV-2 at CDPH in a Biosafety Level 3 laboratory. For cultures, 200 µL of patient specimen was diluted 1:1 with diluent containing 0.75% bovine serum albumin, and 50 µL was added to 8 replicate wells in a 96-well plate containing confluent Vero-81 cells at 37°C with 5% CO_2_. After 1 h, the inoculum was removed and 200 µL of minimum essential medium containing 5% fetal bovine serum and antibiotics was added to each well. Cells were monitored for cytopathic effect. Cells with positive cytopathic effect were tested by rRT-PCR to confirm presence of SARS-CoV-2. Cells with no cytopathic effect or negative rRT-PCR results were passaged after 7 d onto fresh confluent Vero-81 and monitored for an additional 7 d before performing rRT-PCR again. Viral replication in these specimens was defined as a decrease in C_t_ over the culture period.

We used the paired BinaxNOW and rRT-PCR results to calculate the BinaxNOW PPA, NPA, negative predictive value (NPV), and positive predictive value (PPV), using C_t_ <37 to define rRT-PCR–positive specimens. As described in Pilarowski et al. (*5*), we also calculated performance by using C_t_ <30 to define rRT-PCR–positive specimens. The exact binomial method was used to calculate 95% CIs. Comparison of mean C_t_ was performed using the Welch t-test. We performed statistical analyses using R version 4.0.1 (R Foundation for Statistical Computing, https://www.r-project.org).

This activity was reviewed by the Centers for Disease Control and Prevention (CDC) and was conducted consistent with applicable federal law and CDC policy (45 C.F.R. part 46, 21 C.F.R. part 56; 42 U.S.C. §241(d); 5 U.S.C. §552a; 44 U.S.C. §3501 et seq.). In addition, this activity was conducted as part of a COVID-19 project determined to be nonresearch by the California Health and Human Services Agency’s Committee for the Protection of Human Subjects.

## Results

Including testing performed in round 0 and results reported by outside laboratories from staff seeking testing on their own, the cumulative incidence over the course of the outbreak in the entire staff was 62.3% (351/563). A total of 342 different staff participated in testing rounds 1 through 6. These persons ranged in age from 18 to 92 years (median 52 years). Self-reported race and ethnicity produced cell sizes that are too small to report, so only Hispanic ethnicity is presented in this study. Most staff identified as Hispanic (62.0%) (Table 1). Symptoms were reported by 11 different persons at the time of testing, which accounted for 11/769 (1.4%) of collected paired specimens. A total of 6 persons were hospitalized, and 1 of those patients died. The number of staff tested in each round, which varied because of attrition and exclusion of SARS-CoV-2–positive staff from further testing, ranged from 333 persons (round 1) to 57 persons (round 4). The number of rRT-PCR–positive results in each round ranged from 98 (round 1) to 0 (round 4) (Table 2).

**Table 1:** Characteristics of horse racetrack staff providing paired anterior nasal swab specimens for the BinaxNOW rapid antigen test and real-time reverse transcription PCR for coronavirus disease, California, USA, November–December 2020*

Characteristic	rRT-PCR result		Overall
	Detected	Not detected	
Total	127 (100)	215 (100)	342 (100)
Sex			
F	26 (20.5)	62 (28.8)	88 (25.7)
M	101 (79.5)	153 (71.2)	254 (74.3)
Median age (range), y	46 (18–82)	54 (18–92)	52 (18–92)
Age groups, y			
18–44	57 (44.9)	75 (34.9)	132 (38.6)
45–64	56 (44.1)	103 (47.9)	159 (46.5)
>65	14 (11.0)	37 (17.2)	51 (14.9)
Ethnicity			
Hispanic	99 (78.0)	113 (52.6)	212 (62.0)
Non-Hispanic	28 (22.0)	102 (47.4)	130 (38.0)

*Values are no. (%) except as indicated. rRT-PCR, real-time reverse transcription PCR.

**Table 2:** Results of BinaxNOW rapid antigen test compared with real-time reverse transcription PCR for coronavirus disease among all horse racetrack staff undergoing paired testing, California, USA, November–December 2020*

BinaxNOW result	rRT-PCR result		Total
Detected	Not detected
Round 1, n = 333, November 25, 27, 28†			
Detected	40	0	40
Not detected	58	235	293
Total	98	235	333
Round 2, n = 197, December 4			
Detected	12	0	12
Not detected	10	175	185
Total	22	175	197
Round 3, n = 65, December 13			
Detected	2	0	2
Not detected	3	60	63
Total	5	60	65
Round 4, n = 57, December 16			
Detected	0	0	0
Not detected	0	57	57
Total	0	57	57
Round 5, n = 58, December 20			
Detected	0	0	0
Not detected	1	57	58
Total	1	57	58
Round 6, n = 59, December 22			
Detected	1	0	1
Not detected	0	58	58
Total	1	58	59
Overall, N = 769, November 25–December 22			
Detected	55	0	55
Not detected	72	642	714
Total	127	642	769

*Inconclusive (n = 4) and invalid (n = 3) rRT-PCR test results excluded from table. rRT-PCR, real-time reverse transcription PCR. †All employees who had not yet tested positive were meant to return for subsequent testing rounds. However, participant attrition occurred, likely because of employees leaving their jobs or logistical obstacles to being on-site for testing during mass testing days.

In total, 769 valid, paired rRT-PCR and BinaxNOW antigen test results were reported and analyzed. Among all paired testing rounds with rRT-PCR, BinaxNOW produced these results when rRT-PCR tests with C_t_ <37 were considered positive: PPA, 43.3% (95% CI 34.6%–52.4%); NPA, 100% (95% CI 99.4%–100.0%); PPV, 100.0% (95% CI 93.5%–100.0%); and NPV, 89.9% (95% CI 87.5%–92.0%). When only rRT-PCR tests with C_t_ <30 were considered positive, BinaxNOW produced these results: PPA, 55.6% (95% CI 45.2%–65.6%); NPA, 100% (95% CI 99.5%–100%), PPV, 100.0% (95% CI 93.5%–100%); and NPV, 93.8% (95% CI 91.8%–95.5%) (Table 3).

**Table 3:** BinaxNOW rapid antigen test performance compared with real-time reverse transcription PCR for coronavirus disease in using 2 different cycle threshold values to define positive results, California, USA, November–December 2020*

BinaxNOW performance	% (95% CI)	
	C_t_ <37†	C_t_ <30‡
PPA	43.3 (34.6–52.4)	55.6 (45.2–65.6)
NPA	100.0 (99.4–100.0)	100.0 (99.5–100)
PPV	100.0 (93.5–100.0)	100.0 (93.5–100)
NPV	89.9 (87.5–92.0)	93.8 (91.8–95.5)

*Results for 769 paired specimens from 342 horse racetrack staff. C_t_, cycle threshold; NPA, negative percent agreement; NPV, negative predictive value; PPA, positive percent agreement; PPV, positive predictive value; rRT-PCR, real-time reverse transcription PCR. †127 rRT-PCR–positive specimens. ‡100 rRT-PCR–positive specimens.

**Figure 1:**
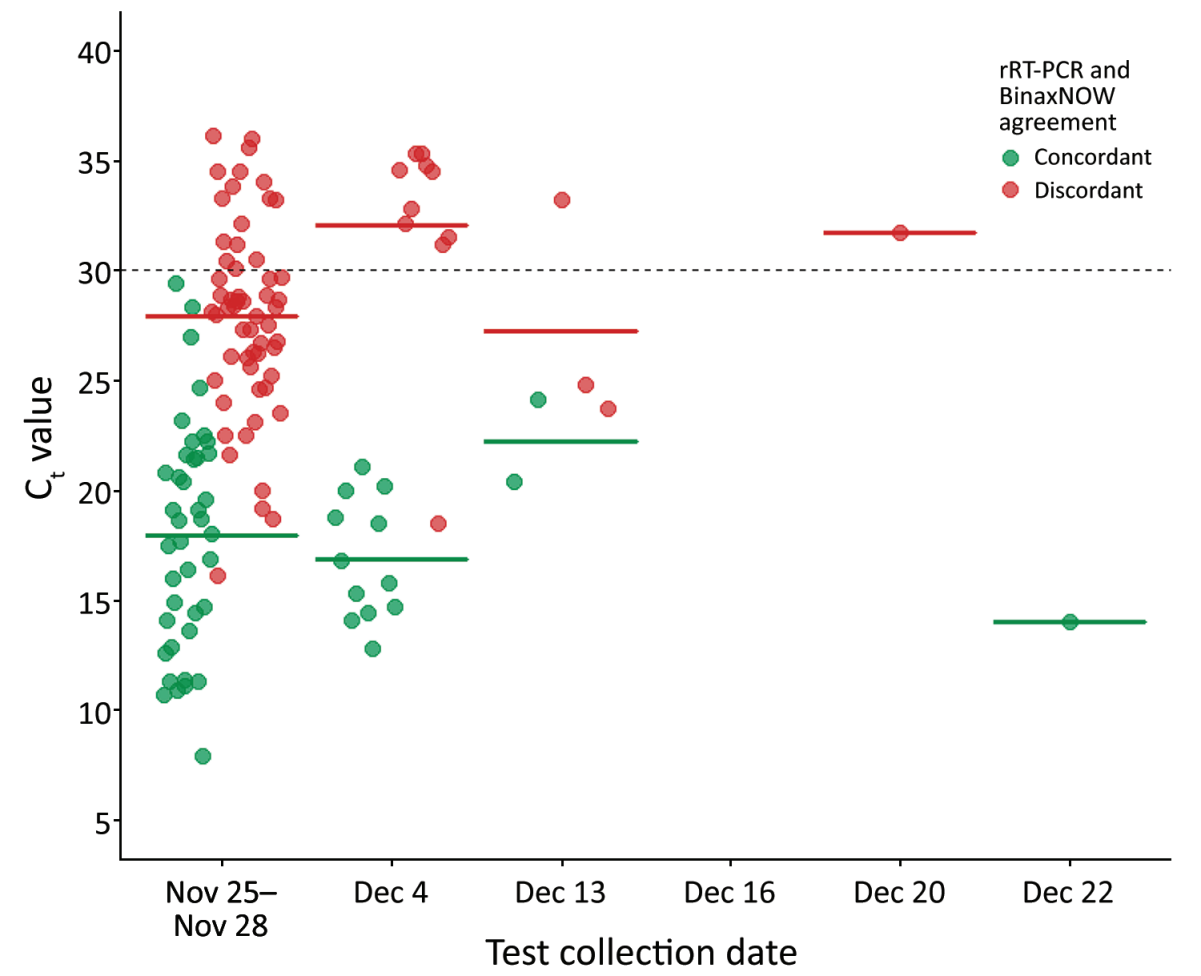
Concordance of BinaxNOW rapid antigen test results with positive rRT-PCR results over 6 testing rounds among staff at a horse racetrack, California, USA, November–December 2020. All rRT-PCR–negative results (n = 642) were concordant with BinaxNOW results, so only rRT-PCR–positive results (n = 127) are shown. Crossbars represent mean C_t_ for the concordant and discordant pair groups in each testing period. The dashed line represents C_t_ = 30. C_t_, cycle threshold; rRT-PCR, real-time reverse transcription PCR.

Of 127 rRT-PCR–positive specimens, BinaxNOW detected 55, did not detect 72 (44 specimens with C_t_ <30, 5 specimens with C_t_ <20, and 6 specimens with positive viral cultures), and produced no false-positive results (Table 3). Among rRT-PCR–positive specimens, those with paired BinaxNOW-positive results had a lower mean C_t_ (17.8) than those with paired BinaxNOW-negative results (28.5) (p < 0.001). No rRT-PCR–positive results with a C_t_ >29.4 were detected by BinaxNOW (Figure 1).

In dual-positive pairs, the median time between rRT-PCR specimen collection date and results reported date was 4 days (range 1–6 days). For BinaxNOW false-negative pairs, the median time between rRT-PCR specimen collection date and results reported date was 5 days (range 1–7 days). In contrast, the 15-minute read time of the BinaxNOW antigen test kit provided results to the facility and LHD the same day as testing.

**Figure 2:**
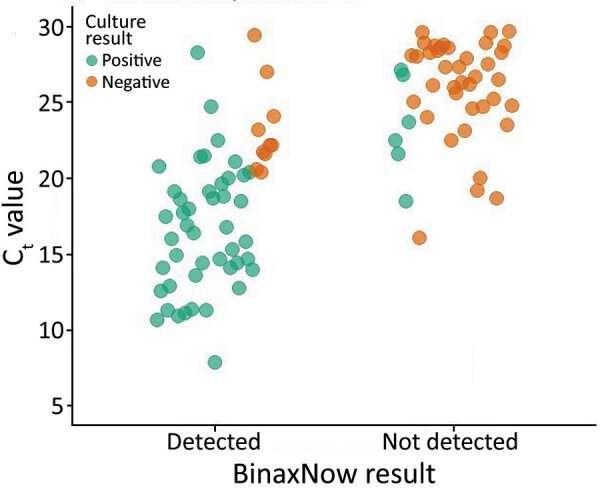
BinaxNOW rapid antigen test results and viral culture results among 100 real-time reverse transcription PCR–positive specimens with cycle threshold <30 among staff at a horse racetrack, California, USA, November–December 2020. Of 51 viral culture–positive specimens, 45 were detected by BinaxNOW (88.2% concordance).

Of the 127 rRT-PCR–positive specimens, we attempted virus isolation and culture for all 100 specimens with C_t_ <30. Of those specimens, 51 resulted in positive virus isolation. Of those culture-positive specimens, 45 (88.2%) were BinaxNOW-positive (Table 4; Figure 2). The mean C_t_ of culture-positive specimens (17.4) was significantly lower than culture-negative specimens (25.5) (p<0.001).

**Table 4:** BinaxNOW rapid antigen test performance compared with viral culture among 100 real-time reverse transcription PCR–positive specimens with cycle threshold <30 from horse racetrack staff, California, USA, November–December 2020

BinaxNOW results	Viral culture results		Total
	Positive	Negative	
Detected	45	10	55
Not detected	6	39	45
Total	51	49	100

## Discussion

In the setting of a nonhealthcare workplace outbreak of COVID-19 with high attack rate (62.3%), we found that BinaxNOW was a useful adjunct to rRT-PCR testing. BinaxNOW showed NPA and PPV of 100%. A total of 55 participants were concordantly identified as positive by BinaxNOW and rRT-PCR, and no false-positive BinaxNOW results were noted. This low false-positive rate is consistent with results from Pilarowski et al. (*5*) that established the updated BinaxNOW card-reading technique used by the racetrack physician in this outbreak. Results of BinaxNOW testing were available the same day, which enabled more rapid identification of infected workers for isolation than reliance on rRT-PCR alone.

Negative BinaxNOW results were less concordant with rRT-PCR results. The PPA of BinaxNOW was 43.0% and the NPV was 89.9%. Real-time RT-PCR confirmation of BinaxNOW negative results identified 72 additional positive specimens. The median time between rRT-PCR specimen collection date and results reported date for these BinaxNOW false-negative specimens was 5 days (range 1–7 days).

Although C_t_ cannot be used to define viral load or infectivity of a given person, C_t_ is inversely related to the amount of target genetic material present in the specimen (*11*). Therefore, the significantly lower mean C_t_ for true-positive BinaxNOW specimens (17.8) compared with false-negative BinaxNOW specimens (28.5) indicates that more viral genetic material was present in those specimens. BinaxNOW demonstrated better concordance with positive viral culture results (88.2%) than with positive rRT-PCR results (43.3%). Positive viral culture is further evidence of the presence of infectious virus, so these findings might indicate that some BinaxNOW false-negative participants were not infectious at the time of specimen collection (i.e., they had low viral RNA load at the beginning or end of their infection trajectory) (*12*). Numerous factors can affect the outcome of a viral culture; therefore, negative culture results do not necessarily mean that no viable virus was present in those specimens, nor that the participants from whom those specimens were collected were not infectious at the time of specimen collection.

With serial BinaxNOW testing, some of the persons with discordant paired results could have tested positive with subsequent BinaxNOW testing. Further studies are needed to determine whether serial rapid antigen testing alone can identify infectious persons as efficiently as rRT-PCR alone or a combination of rRT-PCR and rapid antigen testing (*13*).

The first limitation of our study is that, although other studies have demonstrated differential BinaxNOW test performance in symptomatic and asymptomatic persons (*3*,*6*–*8*), we were unable to examine test performance by symptom status, because symptom reporting might not have been reliable. At the time of specimen collection, only 11 persons reported symptoms to the facility administrative employee registering them for testing. This number conflicts with data previously collected from the racetrack physician as part of a prospective cohort drug trial on this same population which, out of an enrolled cohort of 113 BinaxNOW-positive staff, identified 60 (53%) persons who were symptomatic at the time of testing (*14*). This discrepancy might have resulted from staff feeling less comfortable discussing symptoms with the administrative employee versus the racetrack physician or it could be associated with the incomplete list of COVID-19 symptoms in the administrative employee’s question. It might also reflect a language barrier, because the question about symptoms was asked only in English by the administrative employee. According to onsite interactions with staff and reports from racetrack leadership, many staff were native Spanish speakers, although this language difference was not quantified.

Second, the BinaxNOW tests may have been performed in ambient temperatures below the manufacturer’s recommended range. The BinaxNOW test kit instructions recommend that all test components be at room temperature (15°C–30°C) before use; the mean daily minimum and maximum air temperature recordings from a nearby National Oceanic and Atmospheric Administration weather station in Richmond, CA, on testing days were 7.9°C and 15.1°C (*15*). Performing BinaxNOW tests in the recommended temperature range might have improved performance.

Third, some missing data limit this analysis from encompassing the entire outbreak. The first mass testing dates (round 0) only used rRT-PCR testing, so no comparison with BinaxNOW was possible. Furthermore, each round of testing was intended to capture all staff who had not yet tested positive; however, participant attrition occurred between testing rounds. We attribute this attrition to the logistical obstacles of staff getting to the testing site or to staff leaving their jobs during the outbreak. More complete paired-testing data could have provided better insight as to the usefulness of rapid antigen testing when used for the entire duration of an outbreak.

Our results support considering BinaxNOW-positive employees as infectious without waiting for rRT-PCR confirmation. The rapid turnaround time and high PPV of BinaxNOW enabled some SARS-CoV-2–positive employees to be identified and isolated faster than if rRT-PCR had been used alone. In outbreak situations in which access to laboratory rRT-PCR services is limited, it might be reasonable to act on BinaxNOW-positive results and forgo rRT-PCR confirmation. In contrast, our findings suggest that BinaxNOW negative results in an outbreak investigation should be confirmed with rRT-PCR, because false negatives do occur.

Our results indicate that BinaxNOW performs better at identifying rRT-PCR–positive specimens with lower C_t_ (suggestive of higher viral loads) and positive viral cultures, although these factors are not precise proxies for infectiousness. Real-time RT-PCR remains a more sensitive test for identifying persons that might be infectious, and our results support the current recommendation that rRT-PCR (or another nucleic acid amplification test) should be used in outbreak situations to confirm BinaxNOW-negative results (*2*). Clinical discretion informed by COVID-19 incidence in the relevant population, as well as individual exposure history and symptoms, should be used to determine whether to quarantine persons who test negative for SARS-CoV-2 by BinaxNOW but are awaiting results of rRT-PCR testing (*16*).

## References

[1] Food and Drug Administration. BinaxNOW COVID-19 Ag card (PN 195–000)—instructions for use. 2020 Dec [cited 2021 Mar 15]. https://www.fda.gov/media/141570/download

[2] Centers for Disease Control and Prevention. Interim guidance for antigen testing for SARS-CoV-2. 2020 Dec 5 [cited 2021 Mar 15]. https://www.cdc.gov/coronavirus/2019-ncov/lab/resources/antigen-tests-guidelines.html

[3] James AE, Gulley T, Kothari A, Holder K, Garner K, Patil N. Performance of the BinaxNOW COVID-19 antigen card test relative to the SARS-CoV-2 real-time reverse transcriptase polymerase chain reaction assay among symptomatic and asymptomatic healthcare employees. Infect Control Hosp Epidemiol. 2021; [Epub ahead of print]. 33487197 10.1017/ice.2021.20 PMC7870908

[4] Okoye NC, Barker AP, Curtis K, Orlandi RR, Snavely EA, Wright C, et al. Performance characteristics of BinaxNOW COVID-19 antigen card for screening asymptomatic individuals in a university setting. J Clin Microbiol. 2021;59:e03282–20. 10.1128/JCM.03282-20 33509809 PMC8092740

[5] Pilarowski G, Lebel P, Sunshine S, Liu J, Crawford E, Marquez C, et al. Performance characteristics of a rapid severe acute respiratory syndrome coronavirus 2 antigen detection assay at a public plaza testing site in San Francisco. J Infect Dis. 2021;223:1139–44. 10.1093/infdis/jiaa802 33394052 PMC7799021

[6] Pilarowski G, Marquez C, Rubio L, Peng J, Martinez J, Black D, et al. Field performance and public health response using the BinaxNOW TM Rapid SARS-CoV-2 antigen detection assay during community-based testing. Clin Infect Dis. 2020;ciaa1890; Epub ahead of print. 10.1093/cid/ciaa1890 33367619 PMC7799223

[7] Pollock NR, Jacobs JR, Tran K, Cranston AE, Smith S, O’Kane CY, et al. Performance and implementation evaluation of the Abbott BinaxNOW rapid antigen test in a high-throughput drive-through community testing site in Massachusetts. J Clin Microbiol. 2021;59:e00083–21. 10.1128/JCM.00083-21 33622768 PMC8091851

[8] Prince-Guerra JL, Almendares O, Nolen LD, Gunn JKL, Dale AP, Buono SA, et al. Evaluation of Abbott BinaxNOW rapid antigen test for SARS-CoV-2 infection at two community-based testing sites—Pima County, Arizona, November 3–17, 2020. MMWR Morb Mortal Wkly Rep. 2021;70:100–5. 10.15585/mmwr.mm7003e3 33476316 PMC7821766

[9] Carlsten C, Gulati M, Hines S, Rose C, Scott K, Tarlo SM, et al. COVID-19 as an occupational disease. Am J Ind Med. 2021;64:227–37. 10.1002/ajim.23222 33491195 PMC8014565

[10] Waltenburg MA, Victoroff T, Rose CE, Butterfield M, Jervis RH, Fedak KM, et al.; COVID-19 Response Team. Update: COVID-19 among workers in meat and poultry processing facilities—United States, April–May 2020. MMWR Morb Mortal Wkly Rep. 2020;69:887–92. 10.15585/mmwr.mm6927e2 32644986 PMC7732361

[11] Dahdouh E, Lázaro-Perona F, Romero-Gómez MP, Mingorance J, García-Rodriguez J. C_t_ values from SARS-CoV-2 diagnostic PCR assays should not be used as direct estimates of viral load. J Infect. 2021;82:414–51. 10.1016/j.jinf.2020.10.017 33131699 PMC7585367

[12] Sethuraman N, Jeremiah SS, Ryo A. Interpreting diagnostic tests for SARS-CoV-2. JAMA. 2020;323:2249–51. 10.1001/jama.2020.8259 32374370

[13] Mina MJ, Parker R, Larremore DB. Rethinking Covid-19 test sensitivity—a strategy for containment. N Engl J Med. 2020;383:e120. 10.1056/NEJMp2025631 32997903

[14] Seftel D, Boulware DR. Prospective cohort of fluvoxamine for early treatment of coronavirus disease 19. Open Forum Infect Dis. 2021;8:b050; Epub ahead of print. 10.1093/ofid/ofab050 33623808 PMC7888564

[15] National Oceanic and Atmospheric Administration. Temperature data from the weather station in Richmond, CA, on the following dates: Nov. 25, 27, 28; Dec. 4, 13, 16, 20, 22 [cited 2021 Mar 15]. https://www.noaa.gov

[16] Centers for Disease Control and Prevention. Interim guidance for SARS-CoV-2 testing in non-healthcare workplaces. 2021 Mar 17 [cited 2021 June 11]. https://www.cdc.gov/coronavirus/2019-ncov/community/organizations/testing-non-healthcare-workplaces.html

